# Prevalence of drug-drug interactions in older people before and after hospital admission: analysis from the OPERAM trial

**DOI:** 10.1186/s12877-021-02532-z

**Published:** 2021-10-18

**Authors:** Lorène Zerah, Séverine Henrard, Ingeborg Wilting, Denis O’Mahony, Nicolas Rodondi, Olivia Dalleur, Kieran Dalton, Wilma Knol, Manuel Haschke, Anne Spinewine

**Affiliations:** 1 Clinical Pharmacy Research Group, Université Catholique de Louvain, Louvain Drug Research Institute, Avenue Mounier, 73 bte B1.73.06, 1200 Woluwe-Saint-Lambert, Brussels, Belgium; 2 Institute of Health and Society, Université Catholique de Louvain, Brussels, Belgium; 3 Clinical Pharmacy, University Medical Center Utrecht, Utrecht University, Utrecht, The Netherlands; 4 School of Medicine, Geriatric Medicine, University College Cork, Cork, Ireland; 5 Department of General Internal Medicine, Inselspital, Bern University Hospital, Bern, Switzerland; 6 Institute of Primary Health Care (BIHAM), University of Bern, Bern, Switzerland; 7 Pharmacy, Cliniques universitaires Saint-Luc, Université Catholique de Louvain, Brussels, Belgium; 8 Pharmaceutical Care Research Group, School of Pharmacy, University College Cork, Cork, Ireland; 9 Department of Geriatric Medicine and Expertise Centre Pharmacotherapy in Old Persons, University Medical Center Utrecht, Utrecht University, Utrecht, The Netherlands; 10 Clinical Pharmacology and Toxicology, Department of General Internal Medicine, Inselspital, Bern University Hospital, University of Bern, Bern, Switzerland; 11 Pharmacy Department, Université Catholique de Louvain, CHU UCL Namur, Yvoir, Belgium

**Keywords:** Drug-drug interaction, Prevalence, Polypharmacy, Deprescribing, Older person

## Abstract

**Background:**

Drug-drug interactions (DDIs) are highly prevalent in older patients but little is known about prevalence of DDIs over time. Our main objective was to assess changes in the prevalence and characteristics of drug-drug interactions (DDIs) during a one-year period after hospital admission in older people, and associated risk factors.

**Methods:**

We conducted a sub-study of the European OPERAM trial (OPtimising thERapy to prevent Avoidable hospital admissions in Multimorbid older people), which assessed the effects of a structured medication review (experimental arm) compared to usual care (control arm) on reducing drug-related hospital readmissions. All OPERAM patients (≥70 years, with multimorbidity and polypharmacy, hospitalized in four centers in Bern, Brussels, Cork and Utrecht between December 2016 and October 2018, followed over 1 year) who were alive at hospital discharge and had full medication data during the index hospitalization (at baseline i.e., enrolment at admission, and at discharge) were included. DDIs were assessed using an international consensus list of potentially clinically significant DDIs in older people. The point-prevalence of DDIs was evaluated at baseline, discharge, and at 2, 6 and 12 months after hospitalization. Logistic regression models were performed to assess independent variables associated with changes in DDIs 2 months after baseline.

**Results:**

Of the 1950 patients (median age 79 years) included, 1045 (54%) had at least one potentially clinically significant DDI at baseline; point-prevalence rates were 58, 57, 56 and 57% at discharge, and 2, 6 and 12 months, respectively. The prevalence increased significantly from baseline to discharge (*P* < .001 [significant only in the control group]), then remained stable over time (*P* for trend .31). The five most common DDIs –all pharmacodynamic in nature– accounted for 80% of all DDIs and involved drugs that affect potassium concentrations, centrally-acting drugs and antithrombotics. At 2 months, DDIs had increased in 459 (27%) patients and decreased in 331 (19%). The main factor predictive of a change in the prevalence of DDIs was hyperpolypharmacy (≥10 medications).

**Conclusions:**

DDIs were very common; their prevalence increased during hospitalization and tended to remain stable thereafter. Medication review may help control this increase and minimize the risk of adverse drug events.

**Supplementary Information:**

The online version contains supplementary material available at 10.1186/s12877-021-02532-z.

## Background

Drug-drug interactions (DDIs) occur when two or more drugs interact on a pharmacokinetic and/or a pharmacodynamic level, with the risk of increasing the toxicity or reducing the intended effect of one or more of the involved drugs [1, 2]. Potential DDIs occur when two drugs, known to interact, are prescribed concomitantly; actual DDIs can result in adverse drug events (ADEs) or treatment failure [3–5].

DDIs are highly prevalent in older people [4, 5] as a result of multimorbidity, polypharmacy, age-related changes in pharmacokinetics and pharmacodynamics that increase the complexity of therapeutic management, and treatment by multiple care providers [3, 6–8]. The wide variance in DDI prevalence estimates is a consequence of the considerable heterogeneity in definitions and methods used to identify DDIs, in study populations and in study settings [3–6, 9]. A consensus is needed for identification of DDIs in older people in order to facilitate comparisons across studies [10]. Recently, an international European expert consensus panel used a Delphi process to develop a list of 66 potentially clinically significant DDIs in people aged ≥65 years, which could be used to assess the prevalence of DDIs in epidemiological and interventional studies [11].

Hospital admission in older people with multimorbidity provides an opportunity for medication review, including minimizing the inherent risks of DDIs (current and over time), which is important because DDIs are associated with an increased risk of adverse drug reactions, functional status decline, health services use and mortality in older adults [3, 9, 12–15]. DDIs are responsible for approximately 5% of hospital admissions in older patients [16–18]. However, prescription of new drugs during hospitalization to treat an acute medical problem and/or to prevent readmission may increase the risk of DDIs, particularly if these DDIs persist at and after discharge. To our knowledge, no study previously assessed DDI prevalence over time in older patients, yet such data is important to inform medication review strategies.

The European multicenter, cluster randomized, controlled OPERAM (OPtimising thERapy to prevent Avoidable hospital admissions in Multimorbid older people) trial assessed whether a structured medication review reduced drug-related hospital readmissions in older patients with multimorbidity and polypharmacy compared with usual care [19]. DDIs 2 months after randomization, one of the secondary outcomes measured using the international consensus list, [11] were highly prevalent, with no significant differences between the two arms (59.5% of patients in the intervention and 62.2% in the control arm) [19]. However, no further evaluation of changes in the prevalence of the DDIs was performed in this population.

The main objective of the present study was therefore to evaluate, in patients included in the OPERAM trial, changes in the prevalence of potentially clinically significant DDIs during a one-year period after hospital admission, using the recently developed list of potentially clinically significant DDIs [11]. Secondary objectives were (i) to identify the most common potentially clinically significant DDIs in this population and the drug classes involved, (ii) to determine variables associated with potentially clinically significant DDIs at trial enrolment, and (iii) to identify factors associated with changes in the prevalence of these potentially clinically significant DDIs over time.

## Methods

### OPERAM trial

The OPERAM trial included older (≥70 years) patients with multimorbidity (≥3 chronic medical conditions) and polypharmacy (≥5 chronic medications) [19]. In this trial, 2008 hospitalized patients were enrolled between December 2016 and October 2018 in four medical centers in Bern (Switzerland), Brussels (Belgium), Cork (Ireland) and Utrecht (The Netherlands) [19]. The date of study inclusion (baseline, t_0_) was the first day of the index hospitalization. Follow-up data were collected at the end of the index hospitalization (discharge, t_1_) and via telephone interviews at 2 (t_2_), 6 (t_6_) and 12 (t_12_) months after baseline. A blinded trial team member evaluated and reported all medications taken on those dates, as well as hospitalizations and deaths. Details of the protocol and intervention have been published previously [19–23]. The OPERAM trial received approval from ethics committees at each site. All methods were performed in accordance with the relevant guidelines and regulations.

### Eligibility criteria and follow-up

All the patients included in the OPERAM trial who were alive, had not withdrawn and were not lost to follow-up at hospital discharge, and had full medication data during the index hospitalization were included in this substudy (to be able to measure our main criterion) [19]. Patients with medication data at time t, but with no medication data at t_− 1_, were censored at t_− 1_. For example, a few patients had medication data at 6 months but not at 2 months; these patients were censored at 2 months.

### Drug-drug interactions: prevalence and characteristics

DDIs were identified using recently defined the list of 66 potentially clinically significant DDIs [11]. Throughout the rest of the manuscript, the term “DDI” is used to refer to these potentially clinically significant DDIs.

The drugs most frequently involved in DDIs are cardiovascular, antithrombotic and central nervous system drugs. The expert panel list provides details on the type and mechanism of the DDI (i.e., pharmacodynamic, pharmacokinetic or both), potential harmful effect(s), and management (Additional file 1). An algorithm based on the Anatomical Therapeutic Chemical (ATC) codes of the 66 DDIs was developed (available via https://github.com/agapiospanos/DDI). This algorithm was used to identify DDIs in our patients at the different time points.

In the present study, potential harm resulting from the DDI was evaluated by allocating each DDI to one of the following categories: serious cardiovascular adverse effects; serious neurological adverse effects; bleeding; deterioration of renal function and/or hyperkalemia (including severe myopathy and rhabdomyolysis, which may lead to acute renal failure); hematologic toxicity; and miscellaneous others (Additional file 2).

### Changes in prevalence of DDIs

The prevalence of DDIs at 2 months post-randomization compared to that at baseline was defined as having increased if there was a new prescription giving rise to one or more new DDIs or decreased if deprescription had resulted in removal of one or more DDIs. The 2-month time point was chosen to assess the effect of treatment changes considered during hospitalization, including the weeks following hospital discharge when prescriptions may be adjusted by the general practitioner, for example. This was also the time point selected for several secondary outcomes of the original OPERAM study and therefore all the data were available at this time point [19, 20].

### Statistical analysis

The point-prevalence of DDIs at the different time points (baseline, discharge, 2, 6 and 12 months post-randomization) was evaluated in total (at least one DDI) and for each DDI separately, and then according to the type of interaction (i.e., pharmacokinetic, pharmacodynamic), the potential harm, the randomization arm, and the trial site. The most common DDIs in this population were defined using the 3rd quartile of the most frequent DDIs. McNemar’s test was used to assess differences in prevalence between baseline and discharge. The chi-squared test for trend was used to assess the trend in prevalence between discharge and 1 year after trial enrollment.

Data are presented as median (25–75% interquartile range [IQR]) for continuous variables, and numbers (percentages) for categorical variables. A Mann-Whitney U test or Kruskall-Wallis test was used to compare continuous variables between groups and Pearson’s chi-squared test or Fisher’s exact test to compare categorical variables between groups.

Binary logistic regression models were performed to assess independent variables, present at baseline (age, sex, trial site, medical history, medications, functional autonomy, type of admission, main reason for hospital admission), associated with (i) the presence of at least one DDI at inclusion, and (ii) a change in prevalence of at least one DDI at 2 months (increase or decrease compared to baseline). All variables with a *P* < .15 on univariate analysis were included in the multivariable model. Correlations were assessed between quantitative variables (Pearson coefficient) and qualitative variables (Phi coefficient). The choice between two correlated variables was based on their respective clinical relevance. Missing data were not imputed.

All tests were 2-sided, and a *P* < .05 was considered statistically significant. Statistical analyses were done using R software v4.0.0.

## Results

### Patient characteristics at baseline

**Fig. 1:**
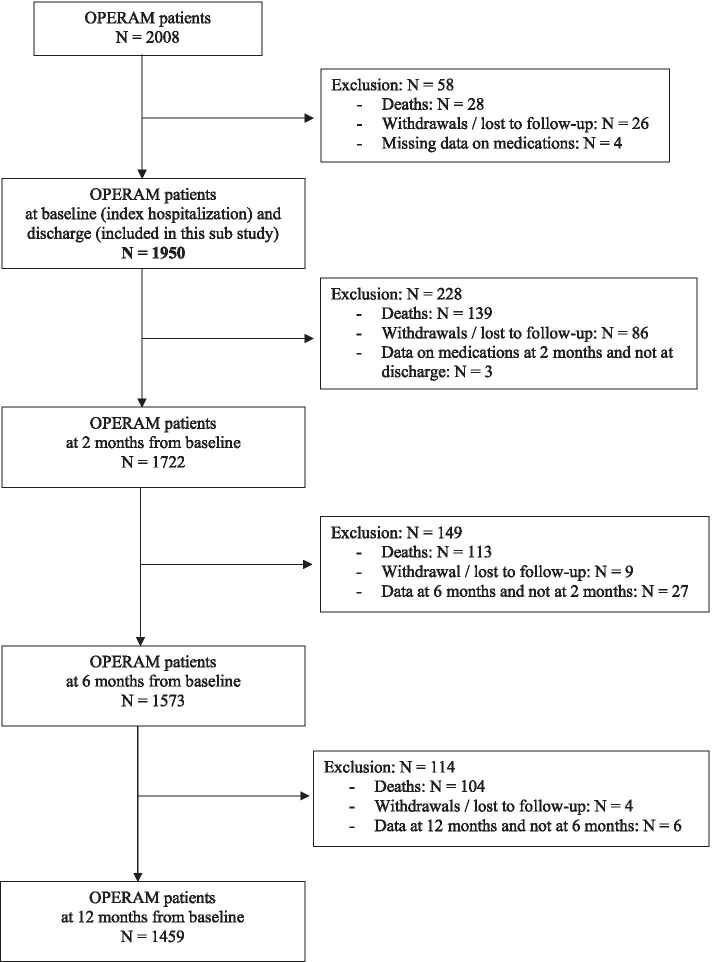
Flow chart

A total of 2008 patients were enrolled in the OPERAM trial, of whom 1950 were alive and had medication data available at discharge and were included in this substudy (Fig. 1). The median age [IQR] was 79 [74–84] years; 1080 patients (55%) were male; the median [IQR] number of drugs per day was 12 [9–16]. Baseline characteristics are given in Table 1 (Additional file 3). [Place of Table 1*]* The median length of stay [IQR] was 7 [3–10] days.

At baseline, 1045 (54%) patients had at least one DDI (Table 1) [median number of DDIs per person [IQR] with at least one DDI: 1[1–2]). Certain medical conditions (depression, coronary artery disease, heart failure, atrial fibrillation, chronic obstructive pulmonary disease [COPD]) and polypharmacy were risk factors for the presence of at least one DDI at baseline, with ORs varying between 1.25 [1.02–1.54] and 2.88 [1.99–4.26] (Table 2). Age over 80 years (80–89 years: 0.79 [0.64–0.97], ≥90 years: 0.65 [0.44–0.96]) and a history of chronic hepatic failure (0.49 [0.31–0.77]) were associated with a lower prevalence of DDIs (Table 2).

**Table 2:** Multivariable analysis of risk factors for the presence of drug-drug interactions at baseline (index hospitalization)

Variables at baseline^a^	OR (95% CI)	***P*** value
**Age (reference: 70–79 years)** - 80–89 years - > 90 years	0.79 [0.64–0.97] 0.65 [0.44–0.96]	.02 .03
**Sex (reference: female)** - male	0.81 [0.66–0.98]	.03
**Medical history of depression**	2.88 [1.99–4.26]	< .001
**Medical history of coronary artery disease**	1.25 [1.02–1.54]	.03
**Medical history of heart failure**	1.60 [1.26–2.02]	< .001
**Medical history of atrial fibrillation**	1.55 [1.26–1.90]	< .001
**Medical history of chronic renal failure**	1.09 [0.87–1.36]	.46
**Medical history of chronic hepatic failure**	0.49 [0.31–0.77]	.002
**Medical history of COPD**	1.91 [1.48–2.47]	< .001
**Hospitalizations during the previous year**	1.17 [0.96–1.42]	.10
**Hyperpolypharmacy**	2.21 [1.77–2.77]	< .001
**Non-independent living**^c^	1.26 [0.98–1.63]	.06
**Main reason for hospital admission (reference: surgical)**^**d**^ - Medical	1.09 [0.82–1.45]	.55

*N* = 1950; AIC = 2490

C statistic: 0.69 IC95% (0.68–0.72).

^a^ Baseline = Index hospitalization

^b^ ≥ 10 drugs par day at admission.

^c^ Non-independent living was defined as living in a nursing home (at least 3 months in the 6 months before the index admission) or being housebound.

^d^Classification coded from the main reasons for hospitalization (free text in the original database)

Abbreviations: *AIC* Akaike’s criterion, *COPD* chronic obstructive pulmonary disease

### Point-prevalence of DDIs over time

**Fig. 2:**
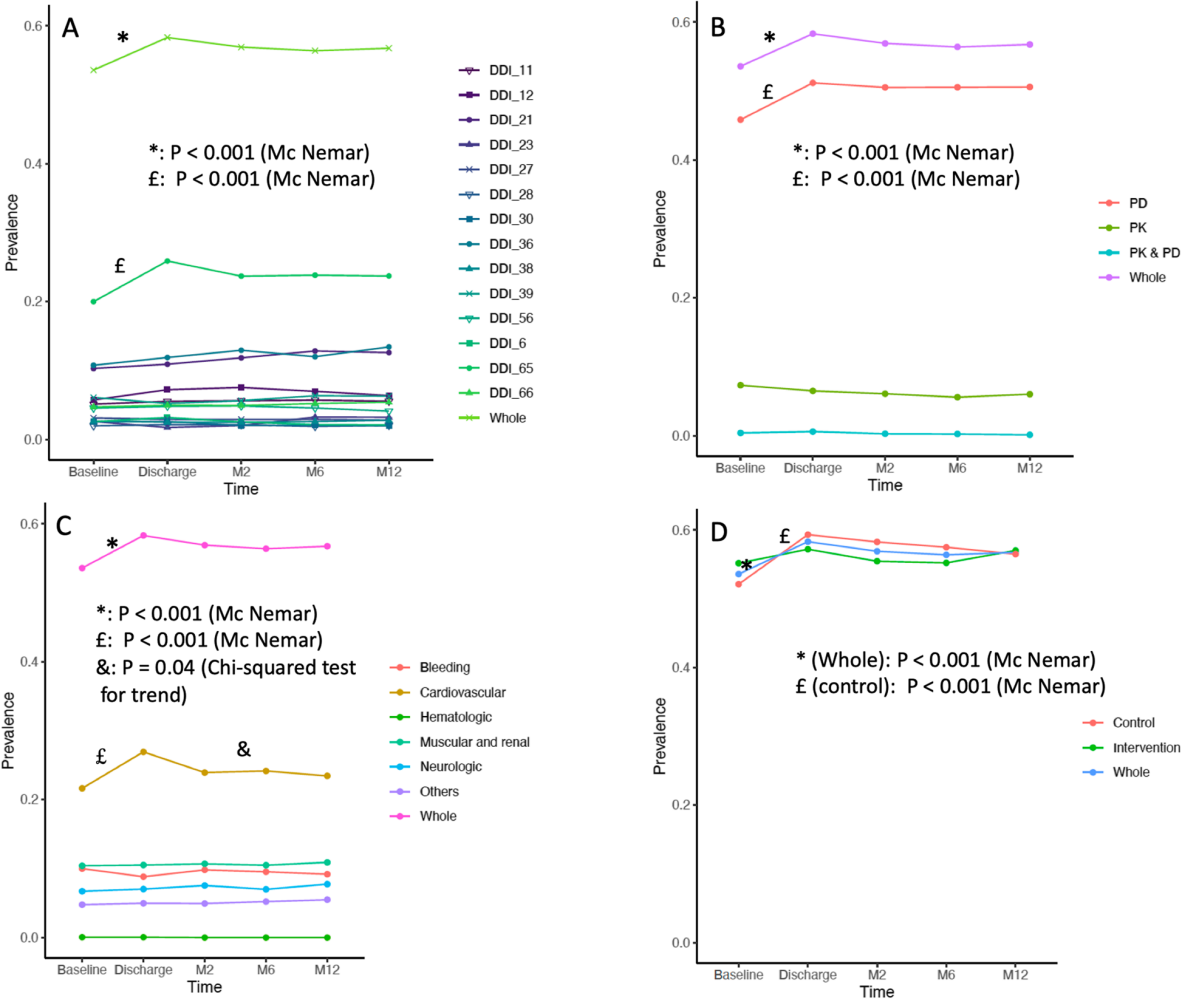
Point-prevalence rates of drug-drug interactions (DDIs) over time. **A**: prevalence in all patients with at least one DDI and for the most frequent DDIs; **B**: prevalence according to pharmacokinetic and pharmacodynamic status; **C**: prevalence according to potential harm; **D**: prevalence according to the OPERAM trial randomization arm. McNemar’s test was used to assess differences in prevalence between baseline and discharge. Chi-squared test for trend was used to assess the prevalence trend between discharge and 1 year after the inclusion. Only *p* values ≤0.05 are specified, other p values are > 0.5 All DDIs, the types of interaction for each DDI, and details on their potential harm are described in Additional file 1**.** DDI 11: oral anticoagulant + an oral non-steroidal anti-inflammatory drug. DDI 12: oral anticoagulant + an antiplatelet drug. DDI 21: concomitant use of ≥2 potassium-sparing drugs. DDI 23: angiotensin-converting enzyme inhibitor or angiotensin II type 1 receptor blockers + an oral non-steroidal anti-inflammatory drug. DDI 27: simvastatin + amlodipine. DDI 28: atorvastatin or simvastatin or lovastatin + amiodarone. DDI 30: calcium channel blocker + a CYP3A4 inhibitor (cytochrome P450). DDI 36: concomitant use of ≥3 centrally-acting drugs. DDI 38: selective serotonin reuptake inhibitor + another serotonergic drug. DDI 39: oral non-steroidal anti-inflammatory drug + selective serotonin reuptake inhibitor or serotonin-norepinephrine reuptake inhibitor. DDI 56: oral or parenteral corticosteroid + an oral non-steroidal anti-inflammatory drug. DDI 6: digoxin + thiazide or loop diuretic. DDI 65: concomitant prescription of ≥2 drugs that reduce potassium. DDI 66: selective serotonin reuptake inhibitor + loop or thiazide diuretic. Abbreviations: M: month, PD: pharmacodynamic, PK: pharmacokinetic, M: month

The point-prevalence rates for at least one DDI were 54, 58, 57, 56 and 57% at baseline, hospital discharge and at 2, 6 and 12 months from the index hospitalization, respectively (Fig. 2A, Additional file 4). The prevalence of DDIs increased significantly during the index hospitalization (McNemar test, *P* < .001), then remained stable over time (Chi-squared test for trend, *P* = .31). The observed increase from baseline to discharge was notable for pharmacodynamic DDIs (but not for pharmacokinetic DDIs) (Fig. 2B), and for DDIs potentially associated with cardiovascular adverse events (Fig. 2C).

The five most prevalent DDIs in our cohort were: DDI 65 (prescription of ≥2 drugs that reduce potassium), DDI 36 (prescription of ≥3 centrally-acting drugs), DDI 21 (prescription of ≥2 potassium-sparing drugs), DDI 12 (oral anticoagulant and antiplatelet) and DDI 39 (oral non-steroidal anti-inflammatory drug and selective serotonin reuptake inhibitor or serotonin-norepinephrine reuptake inhibitor) (Fig. 2A, Additional file 4). These five DDIs accounted for 78% of all DDIs at baseline, 80% at discharge and 83% thereafter. They were all classified as pharmacodynamic interactions (Additional file 1).

The main drug classes involved in the three most frequent DDIs are listed in Additional file 5 (DDI 65: high ceiling diuretics, inhaled beta2 agonists, systemic corticosteroids and contact laxatives; DDI 36: antipsychotics, anxiolytics, hypnotics and sedatives, opioids, antidepressants and antiepileptics; DDI 21: agents acting on the renin-angiotensin system and spironolactone).

As described in Fig. 2D and Additional files 6–7, the prevalence of DDIs during the index hospitalization only increased in patients randomized to the control arm, as compared to the intervention arm, and hospitalized in Bern, as compared to other sites. Patients’ baseline characteristics and types of admission were different across the sites (Additional file 8). Patients in Bern, compared to other sites, had more comorbidities, more polypharmacy, and were generally hospitalized for non-elective reasons (Additional file 8).

### Changes in prevalence of DDIs at 2 months compared to baseline

At 2 months, DDIs had increased in 459 (27%) patients and decreased in 331 (19%) patients compared to at baseline (Additional files 9 and 10). Female sex, hyperpolypharmacy (≥10 drugs per day at baseline), history of depression and heart failure were significantly associated with a decrease in DDIs at 2 months (Table 3). History of atrial fibrillation, coronary heart disease, or cardiac failure and hyperpolypharmacy were significantly associated with an increase in DDIs at 2 months (Table 3).

**Table 3:** Multivariable analysis of factors predictive of change in prevalence (increase or decrease) of drug-drug interactions (DDIs) at 2 months compared to baseline

Variables at baseline^**a**^	OR (95% CI)	***P*** value
**Decrease in DDI**		
**Sex (reference: female)**		
Male	0.76 [0.59–0.98]	.03
**Site (reference: Bern)**		
- Louvain	1.24 [0.87–1.74]	.22
- 1Utrecht	1.12 [0.80–1.55]	.51
- Cork	0.70 [0.47–1.02]	.06
**Medical history of depression**	1.69 [1.14–2.48]	.008
**Medical history of atrial fibrillation**	1.29 [0.99–1.67]	.05
**Medical history of coronary heart disease**	1.17 [0.90–1.53]	.23
**Medical history of heart failure**	1.37 [1.04–1.81]	.03
**Medical history of COPD**	1.21 [0.88–1.63]	.23
**Medical history of cancer**	0.80 [0.58–1.07]	.14
**Hyperpolypharmacy**^**b**^	2.02 [1.44–2.88]	< .001
**Increase in DDI**		
**Site (reference: Bern)**		
- Louvain	0.72 [0.50–1.02]	.07
- Utrecht	0.69 [0.50–0.95]	.02
- Cork	0.53 [0.37–0.74]	< .001
**Medical history of dementia**	1.18 [0.75–1.85]	.46
**Medical history of depression**	1.30 [0.88–1.90]	.17
**Medical history of atrial fibrillation**	2.01 [1.59–2.55]	< .001
**Medical history of coronary heart disease**	1.29 [1.01–1.64]	.04
**Medical history of cardiac failure**	1.79 [1.39–2.30]	< .001
**Medical history of chronic renal failure**	0.85 [0.65–1.10]	.23
**Medical history of bleeding**	1.29 [0.93–1.77]	.11
**Hyperpolypharmacy**^**b**^	1.74 [1.29–2.37]	< .001
**ADL**	0.95 [0.87–1.03]	.19
**Main reason for hospital admission (reference: surgical)**		
- Medical	1.32 [0.90–1.97]	.16

Deprescription: *N* = 1722, AIC = 1647.9, C statistics: 0.63 IC95% (0.61–0.67).

Prescription: *N* = 1701, AIC = 1857, C statistics: 0.69 IC95% (0.67–0.73).

^a^ Baseline = Index hospitalization

^b^ ≥ 10 drugs par day at admission

Univariate analyses are described in Additional files 9 and 10

Abbreviations: *AIC* Akaike’s criterion, *ADL* activities of daily living, *COPD* chronic obstructive pulmonary disease

**Table 1:** Demographic data and baseline characteristics of patients included in the cohort, stratified by drug-drug interaction (DDI) status at baseline

	Total ***N*** = 1950	At least one DDI at baseline^a^ ***N*** = 1045	No DDI at baseline^a^ ***N*** = 905	Missing values	***P v***alue
**Age (years)**					
Median (IQR)	79 [74–84]	78 [74–83]	79 [74–85]	0	.13 .25
70–79	1053 (54)	582 (56)	471 (52)		
80–89	757 (39)	393 (37)	364 (40)		
> 90	140 (7)	70 (7)	70 (8)		
**Male**	1080 (55)	558 (53)	522 (58)	0	.06
**Trial site**					
Louvain, Belgium	385 (20)	206 (20)	179 (20)	0	.25
Cork, Ireland	328 (17)	171 (16)	157 (17)		
Utrecht, The Netherlands	433 (22)	250 (24)	183 (20)		
Bern, Switzerland	804 (41)	418 (40)	386 (43)		
**Medical history**					
Dementia	116 (6)	65 (6)	51 (6)	0	.65
Depression	173 (9)	132 (13)	41 (5)		< .001
Stroke	410 (21)	207 (20)	203 (22)		.17
Hypertension	1282 (66)	675 (65)	607 (67)		.27
Atrial fibrillation	697 (36)	436 (42)	261 (29)		< .001
Diabetes	628 (32)	346 (33)	282 (31)		.38
Coronary artery disease	661 (34)	385 (37)	276 (31)		.004
Heart failure	500 (26)	333 (32)	167 (19)		< .001
Chronic renal failure	522 (27)	302 (29)	220 (24)		.03
Chronic hepatic failure	98 (5)	41 (4)	57 (6)		.02
COPD	375 (19)	258 (25)	117 (13)		< .001
Cancer	482 (25)	249 (24)	233 (26)		.35
Bleeding	250 (13)	137 (13)	113 (12)		.73
Thromboembolic disease	236 (12)	133 (13)	103 (11)		.40
Number of comorbidities	11 [8–16]	11 [8–16]	11 [8–15]		.002
**Medications on index admission**					
Number of drugs per day	12 [9–16]	14 [11–18]	11 [8–14]	0	< .001
Hyperpolypharmacy^b^	1458 (75)	873 (83)	585 (65)		
**Any hospitalizations during the previous year** Number of hospitalizations during the previous year	986 (51) 1 [0–1]	566 (54) 1 [0–2]	420 (46) 0 [0–1]	0	.001 < .001
**Not living independently**^**c**^	375 (19)	222 (21)	153 (17)	0	.02
**ADL6 score**	5.5 [4.5–6.0]	5.5 [4.5–6.0]	5.5 [4.5–6.0]	32	.61
**Education level**					
Less than high school education	573 (30)	326 (32)	247 (28)	24	.16
High-school degree	886 (46)	463 (45)	423 (47)		
Post-secondary degree	467 (24)	243 (23)	224 (25)		
**Type of admission**					
Elective^**d**^	473 (24)	243 (23)	230 (25)	0	.29
Non-elective	1477 (76)	802 (77)	675 (75)		
**Main reason for hospital admission**					
Surgical	257 (13)	120 (11)	137 (15)	0	.02
Medical	1693 (87)	925 (89)	768 (85)		

^a^ Baseline = Index hospitalization

^b^ ≥ 10 drugs par day at admission

^c^ Non-independent living was defined as living in a nursing home (at least 3 months in the 6 months before the index admission) or being housebound

^d^ Elective procedure for a pre-existing condition

Data are median [P25; P75] or n (%). Comparison between the two groups using Mann-Whitney U test for quantitative variables and chi-square test or Fisher’s exact test for qualitative variables

Abbreviations: *COPD* chronic obstructive pulmonary disease

## Discussion

### Key findings

In a European cohort of older patients with multimorbidity and polypharmacy, the prevalence of potentially clinically significant DDIs was high (54%) at hospital admission. Patients with certain medical conditions, such as depression, coronary artery disease, heart failure, atrial fibrillation and COPD, and those with polypharmacy were more likely to have at least one DDI at baseline. The top five most frequent potentially clinically significant DDIs (drugs that reduce potassium [diuretics, inhaled beta2 agonists, systemic corticosteroids], centrally-acting drugs [psychotropics, antidepressants, opioids, antiepileptics], potassium-sparing drugs [angiotensin-converting enzyme, angiotensin II type 1 receptor blockers, spironolactone] and antithrombotics] comprised 80% of all potentially clinically significant DDIs. These five potentially clinically significant DDIs were all classified as pharmacodynamic interactions mainly causing cardiovascular adverse events. The prevalence of potentially clinically significant DDIs increased significantly at discharge and then remained stable over the subsequent 12 months (i.e., did not return to the baseline prevalence level).

### Comparisons with previous studies

The high prevalence of DDIs in our cohort aligns with the in-patient and out-patient prevalences reported in the literature for comparable populations (geriatric outpatient cohort: 44.5% [24] and 58.3% [25]; geriatric inpatient cohort: 60.5% [26]). The drug classes most frequently involved in DDIs in our cohort were also the same as those previously reported [1, 4, 16, 17, 26–29]. These drugs are often prescribed to older patients to treat common age-related conditions, particularly cardiovascular and neurological conditions. Consistent with our results, Vonbach et al. reported that more than 70% of all major and moderately severe potential DDIs were pharmacodynamic interactions [30].

In line with our results, two studies reported that hospitalization was associated with an increase in DDIs in older patients, [26, 30] one of which reported that almost half of the DDIs at hospital discharge were incurred during hospitalization [30]. However, and this is the originality of our study, none of the published studies assessed DDIs over time to describe trends after discharge in older patients. Changes in the prevalence of DDIs over time, with a higher prevalence at discharge than at baseline, and a decrease after hospital discharge but not to baseline levels, were noted particularly for DDI 65 (concomitant prescription of ≥2 drugs that reduce potassium), which includes cardiovascular and respiratory drugs (high ceiling diuretics and inhaled beta2 agonists). This trend has already been described in patients with coronary heart disease and COPD, [31] conditions for which these drugs are indicated. In addition, factors associated with an increase in DDIs at 2 months were similar to those previously reported in the literature, especially polypharmacy [2–4, 7, 32]. The fact that the patients included in Bern had more comorbidities, were more likely to have polypharmacy, and were generally hospitalised for non-elective reasons, may explain the differences in trends across trial sites.

DDIs are often classified according to their potentially harmful effects, but no study has reported the prevalence of DDIs according to whether or not they may be appropriate. Evaluation of appropriateness of a DDI often requires an individual, case-by-case assessment, which is not possible on a large database. However, from the list of 66 DDIs, some can be considered inappropriate in many cases (“avoid concurrent use”), such as DDI 32, “beta-blocker and verapamil or diltiazem” (potentially serious cardiovascular adverse effects in particular in patients predisposed to heart failure); DDI 36, “concomitant use of ≥3 centrally-acting drugs” (increased risk of falls, fracture, impaired cognition); and DDI 63, “tamoxifen and paroxetine or fluoxetine or bupropion” (risk of sudden death – ventricular arrhythmias, torsade de pointes). Most drugs in these DDI categories were rarely prescribed in our cohort with the exception of DDI 36. Other DDIs may be appropriate, or even intentional, in some cases if preventive measures are taken (e.g., dose adaptation, close monitoring, patient education), for example, for DDI 12, “oral anticoagulant and antiplatelet drug”; DDI 65, “concomitant prescription of ≥ 2 drugs that reduce potassium”; and DDI 66: “selective serotonin reuptake inhibitor and loop or thiazide diuretic”. DDI 65 prescribed in acute clinical situations, such as cardiac failure or COPD exacerbation, may, for example, be appropriate if serum potassium concentrations are monitored closely. Similarly, DDI 12 is only appropriate in certain specific clinical situations (e.g., atrial fibrillation and recent acute coronary syndrome) and for a fixed duration [33]. The fact that 57% of patients in the experimental arm (i.e., patients for whom medication review was performed by a physician and pharmacist) had at least one DDI at hospital discharge suggests that most of these DDIs were considered appropriate in these patients and thus did not require drug discontinuation or modification after the medication review. Only the patients in the control arm had an increase in the prevalence of DDI 36 from baseline to discharge, which may explain why there was a significant increase in the prevalence of DDIs during hospitalization only in patients in the control arm. These data suggest that frequent medication reviews for those admitted to hospital, not only during hospital admission, but also after discharge, can contribute to reducing the risk of inappropriate DDIs.

### Strength and limitations

To our knowledge, this is the first international, multicenter study to report data on the prevalence of DDIs at hospital admission and at different time points over a one-year follow-up period in an older population with multimorbidity. To identify the DDIs, we used for the first time a new list of 66 potentially clinically significant DDIs developed by an international consensus panel [11].

This study has some limitations. First, we were unable to distinguish between appropriate and inappropriate DDIs (which would require detailed information on, for example, dose adaptations, indications, monitoring, patient specifics). Second, because of a lack of statistical power, we did not assess ADEs and drug-related hospital admissions associated with DDIs. This information would have enabled us to evaluate actual DDIs resulting from potential DDIs. As reported in the literature, it is therefore likely that we have overestimated the true clinical significance of the DDIs [3]. Indeed, studies that focused on actual DDIs reported lower prevalences in ambulatory settings (9.5 and 25.5%) [24, 34] as well as for in-patients (8.8%) [35]. Nevertheless, the presence of a potentially clinically significant DDI remains a strong signal that should alert the prescriber to the need for greater patient monitoring and education.

### Perspectives

It seems important to help clinicians identify potentially harmful DDIs more effectively, in particular the five most prevalent, to improve their assessment of risk-benefit ratios in individual patients. Risk minimization measures for prescription of psychotropic drugs, antithrombotics and drugs that reduce or increase potassium concentrations should be deployed.

The next logical step would be to assess the clinical impact of these DDIs, especially of the most common ones. To this end, a large scale cohort study from healthcare databases could be carried out. Better identification of the impact of DDIs is important for clinicians, researchers and health policy decision-makers to plan for safer healthcare in ageing societies.

## Conclusions

In a European cohort of older patients with multimorbidity and polypharmacy, we evaluated, for the first time, the prevalence of DDIs over a 12-month interval using a new list of 66 potentially clinically significant DDIs in older patients. The prevalence of DDIs in hospital and after discharge was high, with more than half the cohort having at least one DDI within their medication regimen. Prescriptions resulting in additional DDIs occurred during the index hospital stay (in particular in the control group without medication review) and tended to remain stable thereafter. Many of these DDIs were probably appropriate/deliberate at a patient level, if adequate management measures (mainly monitoring and patient education) are taken. Efforts should be directed at identifying inappropriate/potentially dangerous DDIs. Medication review during the hospital stay should help achieve this.

Further evaluation of the performance of the DDI list in terms of its association with clinical outcomes and identification of associated factors will be useful to further refine the list and identify inappropriate DDIs and avoidable ADEs.

## Supplementary Information

**Additional file 1 **Additional file 1 List of potentially clinically significant drug-drug interactions (DDIs) in older people (*n* = 66). Additional file 2 Classification of drug-drug interactions (DDIs) according to their potential harm. Additional file 3. International Classification of Diseases, 10th revision (ICD-10) codes used to identify comorbid conditions during the index hospitalization. Additional file 4. Prevalence (%) of all drug-drug interactions (DDIs) over time. Additional file 5. The three most common drug-drug interactions (DDIs) in this cohort and medication classes involved. Additional file 6. Prevalence of drug-drug interactions (DDIs) during follow-up (at least one DDI (“whole”) and DDIs belonging to the 3rd most frequent quartile (prevalence ≥1.5%)). A: in the experimental arm, B: in the control arm. Additional file 7. Prevalence of drug-drug interactions (DDIs) during follow-up: at least one DDI for the whole cohort (“whole”) and according to site. Additional file 8. Demographic data and baseline characteristics of older patients included in the cohort and stratified by site. Additional file 9. Demographic data and baseline characteristics of older patients present at 2 months and stratified by DDI decrease status at 2 months compared to baseline. Additional file 10. Demographic data and baseline characteristics of older patients included in our cohort and stratified by DDI increase status at 2 months compared to baseline

## Data Availability

Data for this study will be made available to others in the scientific community upon request after the publication date. Data will be made available for scientific purposes of researchers whose proposed use of the data has been approved by a publication committee. Data and documentation will be made available via a secure file exchange platform after approval of proposal and a data transfer agreement is signed (which defines obligations that the data requester must adhere to with regard to privacy and data handling). Partially de-identified participant data limited to the data used for this work will be made available, along with a data dictionary and annotated case report forms. For data access, please contact Pr Anne Spinewine: anne.spinewine@uclouvain.be.

## Abbreviations

ADE: Adverse drug events
ATC: Anatomical Therapeutic Chemical
COPD: Chronic obstructive pulmonary disease
DDI: Drug-drug interaction
OPERAM: OPtimising thERapy to prevent Avoidable hospital admissions in Multimorbid older people
