# Supplementary material for: Prevalence of drug-drug interactions in older people before and after hospital admission: analysis from the OPERAM trial
Source: BMC Geriatr. 2021 Oct 18;21:571. doi: 10.1186/s12877-021-02532-z (PMC8524798; doi:10.1186/s12877-021-02532-z)
Supplement: Supplementary file 1 — Additional file 1 Additional file 1 List of potentially clinically significant drug-drug interactions (DDIs) in older people (n = 66). Additional file 2 Classification of drug-drug interactions (DDIs) according to their potential harm. Additional file 3. International Classification of Diseases, 10th revision (ICD-10) codes used to identify comorbid conditions during the index hospitalization. Additional file 4. Prevalence (%) of all drug-drug interactions (DDIs) over time. Additional file 5. The three most common drug-drug interactions (DDIs) in this cohort and medication classes involved. Additional file 6. Prevalence of drug-drug interactions (DDIs) during follow-up (at least one DDI (“whole”) and DDIs belonging to the 3rd most frequent quartile (prevalence ≥1.5%)). A: in the experimental arm, B: in the control arm. Additional file 7. Prevalence of drug-drug interactions (DDIs) during follow-up: at least one DDI for the whole cohort (“whole”) and according to site. Additional file 8. Demographic data and baseline characteristics of older patients included in the cohort and stratified by site. Additional file 9. Demographic data and baseline characteristics of older patients present at 2 months and stratified by DDI decrease status at 2 months compared to baseline. Additional file 10. Demographic data and baseline characteristics of older patients included in our cohort and stratified by DDI increase status at 2 months compared to baseline [file 12877_2021_2532_MOESM1_ESM.pdf]

PREVALENCE OF DRUG-DRUG INTERACTIONS IN OLDER PEOPLE BEFORE AND AFTER HOSPITAL ADMISSION: ANALYSIS FROM THE OPERAM TRIAL

Lorène Zerah MD PhD[1], Séverine Henrard PhD[1,2], Ingeborg Wilting PhD[3], Denis O'Mahony MD PhD[4], Nicolas Rodondi MD PhD[5,6], Olivia Dalleur PhD[1,7], Kieran Dalton PhD[8], Wilma Knol MD PhD[9], Manuel Haschke MD[10], Anne Spinewine PhD[1,11]

1. Clinical Pharmacy Research Group, Université Catholique de Louvain, Louvain Drug Research Institute, Brussels, Belgium.
2. Institute of Health and Society, Université Catholique de Louvain, Brussels, Belgium.
3. Clinical Pharmacy, University Medical Center Utrecht, Utrecht University, Utrecht, The Netherlands
4. School of Medicine, Geriatric Medicine, University College Cork, Cork, Ireland
5. Department of General Internal Medicine, Inselspital, Bern University Hospital, University of Bern, Bern, Switzerland
6. Institute of Primary Health Care (BIHAM), University of Bern, Bern, Switzerland
7. Pharmacy, Cliniques universitaires Saint-Luc, Université Catholique de Louvain, Brussels, Belgium
8. Pharmaceutical Care Research Group, School of Pharmacy, University College Cork, Cork, Ireland.
9. Department of Geriatric Medicine and Expertise Centre Pharmacotherapy in Old Persons, University Medical Center Utrecht, Utrecht University, Utrecht, The Netherlands
10. Clinical Pharmacology and Toxicology, Department of General Internal Medicine, Inselspital, Bern University Hospital, University of Bern, Switzerland
11. Pharmacy Department, Université Catholique de Louvain, CHU UCL Namur, Yvoir, Belgium

Correspondence to: Dr Lorène Zerah

Louvain Drug Research Institute, Clinical Pharmacy Research Group, Avenue Mounier, 73 bte B1.73.06, 1200 Woluwe-Saint-Lambert, Belgique

Lorene.zerah@uclouvain.be

ORCID no: 0000-0002-3474-2200

Additional files:

Additional file 1. List of potentially clinically significant drug-drug interactions (DDIs) in older people (*n*=66)

Additional file 2. Classification of drug-drug interactions (DDIs) according to their potential harm

Additional file 3. International Classification of Diseases, 10th revision (ICD-10) codes used to identify comorbid conditions during the index hospitalization

Additional file 4. Prevalence (%) of all drug-drug interactions (DDIs) over time

Additional file 5. The three most common drug-drug interactions (DDIs) in this cohort and medication classes involved

Additional file 6. Prevalence of drug-drug interactions (DDIs) during follow-up (at least one DDI (« whole ») and DDIs belonging to the 3rd most frequent quartile (prevalence ≥ 1.5%)) : A: in the experimental arm , B: in the control arm

Additional file 7. Prevalence of drug-drug interactions (DDIs) during follow-up: at least one DDI for the whole cohort ("whole") and according to site

Additional file 8. Demographic data and baseline characteristics of older patients included in the cohort and stratified by site

Additional file 9. Demographic data and baseline characteristics of older patients present at 2 months and stratified by DDI decrease status at 2 months compared to baseline

Additional file 10. Demographic data and baseline characteristics of older patients included in our cohort and stratified by DDI increase status at 2 months compared to baseline

Additional file 1. List of potentially clinically significant drug-drug interactions (DDIs) in older people (*n*=66)[1]

DDI-number	Drug-drug interaction pairs	Type of DDI†	Potential harm	Management
DDI-1	digoxin + amiodarone	PK + PD	Digoxin toxicity, that may lead to potentially fatal cardiac arrhythmia	- Monitor serum digoxin levels closely and adjust dosage accordingly<br>- Advise patients to promptly report any signs of digoxin toxicity such as nausea, vomiting, anorexia, visual disturbances, slow pulse/bradycardia, or irregular heartbeat/arrhythmia<br>- DDI-1: Reduce the digoxin dosage by one-third to one-half<br>- DDI-2: Reduce the digoxin dosage by one-third to one-half<br>- DDI-4: Reduce the digoxin dosage by one-half<br>- DDI-5: Substitute with non-macrolide antibiotic or reduce digoxin dosage by one-third to one-half<br>- DDI-6: Closely monitor serum levels of digoxin, potassium and magnesium
DDI-2	digoxin + verapamil or diltiazem	PK + PD		
DDI-3	digoxin + propafenone	Unknown		
DDI-4	digoxin + quinidine	PK		
DDI-5	digoxin + some macrolides (i.e. erythromycin or clarithromycin or azithromycin or roxithromycin or telithromycin)	PK		
DDI-6	digoxin + thiazide or loop diuretic	PD		
DDI-7	vitamin K antagonist + a fibrate	PK + PD	Bleeding	- Monitor the INR closely and adjust the vitamin K antagonist dosage accordingly<br>- Advise patients to report any signs of excessive anticoagulation such as unusual or prolonged bleeding, bruising, vomiting, change in stool or urine colour, headache, dizziness or weakness<br>- DDI-7: Reduce the vitamin K antagonist dosage by one-third to one-half<br>- DDI-8: Substitute with a non-interacting gastroprotective drug (e.g. PPI like pantoprazole or another H_2 antagonist)<br>- DDI-10: Reduce the vitamin K antagonist dosage by one-quarter to one-half
DDI-8	vitamin K antagonist + cimetidine	PK		
DDI-9	vitamin K antagonist + metronidazole	PK		
DDI-10	vitamin K antagonist + amiodarone	PK		
DDI-11	oral anticoagulant (i.e. vitamin K antagonist or factor Xa inhibitor or direct thrombin inhibitor) + an oral NSAID	PD	Bleeding, gastrointestinal bleeding and toxicity (i.e. inflammation, ulceration and perforation)	- Advise patients to promptly report any signs of excessive anticoagulation such as unusual or prolonged bleeding, bruising, vomiting, change in stool or urine colour, headache, dizziness or weakness<br>- DDI-11: Consider the addition of a PPI or H2 antagonist during treatment with NSAID

DDI-12	oral anticoagulant + an antiplatelet drug (including aspirin)	PD	Bleeding	
DDI-13	vitamin K antagonist + trimethoprim/sulfamethoxazole	PK + PD	Bleeding	- Monitor the INR closely and adjust the vitamin K antagonist dosage accordingly<br>- Advise patients to promptly report any signs of excessive anticoagulation such as unusual or prolonged bleeding, bruising, vomiting, change in stool or urine colour, headache, dizziness or weakness<br>- DDI-13: Substitute with another antibiotic
DDI-14	vitamin K antagonist + a quinolone	Unknown		
DDI-15	vitamin K antagonist + a macrolide	PK + PD		
DDI-16	dabigatran + a P-gp inhibitor (ketoconazole, itraconazole, verapamil, quinidine, amiodarone, dronedarone, ciclosporin, clarithromycin, erythromycin, ritonavir)	PK	Bleeding	- Advise patients to promptly report any signs of excessive anticoagulation such as unusual or prolonged bleeding, bruising, vomiting, change in stool or urine colour, headache, dizziness or weakness<br>- Specific recommendations for management may differ depending on: presence of risk factors (including renal failure), indication, interacting drug. Refer to appropriate literature and SmPC.<br>- DDI-16: Not recommended with ketoconazole, itraconazole, ciclosporin, dronedarone, ritonavir. For other interacting drugs, use with caution and/or reduce dosage.<br>- DDI-17: Reduce dosage or use with caution.<br>- DDI-18: Not recommended with azoles, ritonavir, dronedarone. For other interacting drugs, avoid use or use with caution<br>- DDI-19: Not recommended with azoles, ritonavir. For other interacting drugs, avoid use, reduce dosage and/or use with caution
DDI-17	edoxaban + a P-gp inhibitor (same list as for DDI-16)	PK		
DDI-18	rivaroxaban + a P-gp inhibitor or a CYP3A4-inhibitor ‡ (ketoconazole, itraconazole, voriconazole, posaconazole, fluconazole, diltiazem, verapamil, quinidine, dronedarone, amiodarone, ciclosporin, , clarithromycin, erythromycin, ritonavir)	PK		
DDI-19	apixaban + a P-gp inhibitor or a CYP3A4-inhibitor ‡	PK		
DDI-20	antiplatelet drug (including aspirin) + oral NSAID	PD	Bleeding, gastrointestinal toxicity (inflammation,	- Consider the addition of gastroprotective drugs (e.g. PPI)<br>- Advise patients to promptly report any signs of ulceration and bleeding such as abdominal pain, bloating, sudden dizziness or light-headedness, nausea, vomiting, hematemesis, anorexia, and melena

			ulceration, perforation)<br>Decreased cardioprotective effect with aspirin	- In order to preserve the cardioprotective effect of low-dose aspirin, administer the latter at least 2 hours before or at least 8h after NSAID intake
DDI-21	concomitant use of ≥ 2 potassium-sparing drugs (i.e. amiloride, triamterene, eplerenone, spironolactone, ACE inhibitors, ARBs, NSAIDs, trimethoprim/sulfamethoxazole)	PD	Hyperkalaemia	- Closely monitor patients for serum potassium levels and renal function<br>- Educate patients about the potential danger of excessive potassium in the diet and advise them to promptly report any signs of hyperkalaemia such as nausea, vomiting, weakness, listlessness, tingling of the extremities, paralysis, confusion, weak pulse, and a slow or irregular heartbeat<br>- DDI-21: Extra caution is required in patients with moderate renal impairment, diabetes, severe or worsening heart failure, dehydration, or concomitant therapy with other agents that increase serum potassium such as beta-blockers, ciclosporine, heparin, tacrolimus, and trimethoprim. Avoid concurrent use in patients with severe renal impairment (CrCl < 30 ml/min)
DDI-22	ACE inhibitor or ARB or a potassium-sparing diuretic + a potassium supplement	PD		
DDI-23	ACE inhibitor or ARB + an oral NSAID	PD	Deterioration of renal function and hyperkalaemia<br>Altered blood pressure control	- Keep the use of NSAIDs to a minimum in patients on antihypertensives, especially in those with blood pressures that are relatively high, as well as in those with high salt intake<br>- Monitor patient for altered blood pressure control and for renal function<br>- Ensure adequate hydration, avoiding dehydration or fluid overload
DDI-24	diuretic + oral NSAID	PD	Deterioration of renal function, hyperkalaemia and congestive heart failure<br>Altered blood	- Keep the use of NSAID to a minimum in patients taking diuretics, especially in those with blood pressures that are relatively high, as well as in those with high salt intake or with congestive heart failure<br>- Monitor patients for signs of worsening renal function and assure diuretic efficacy, including appropriate effects on blood pressure<br>- Ensure adequate hydration, avoiding dehydration or fluid overload

			pressure control	
DDI-25	statin + gemfibrozil	PK	Severe myopathy and rhabdomyolysis which may lead to acute renal failure and death	- Discontinue statin therapy if creatine kinase is markedly elevated in the absence of strenuous exercise or if myopathy is otherwise suspected or diagnosed<br>- Advise patients to promptly report any unexplained muscle pain, tenderness or weakness, particularly if accompanied by fever, malaise, dark-coloured urine<br>- DDI-25: Contraindicated in a number of conditions considered to be risk factors for myopathy (i.e. renal impairment, hypothyroidism). Reduce the statin dosage to the lowest effective dose and consider using a fibrate other than gemfibrozil. If maintained, gemfibrozil dosage should not exceed 10mg daily<br>- DDI-26,27, 28: Consider safer alternatives not metabolized by CYP3A4 (e.g. fluvastatin, pravastatin or rosuvastatin).<br>- DDI-26,27, 28, 29: Reduce the dosage of involved statins to the lowest effective dose –do not exceed 20mg simvastatin and 40mg lovastatin daily<br>- DDI-29: Substitute with a non-interacting antibiotic or temporarily withdraw the statin as long as macrolide antibiotics are required, except if benefits outweigh risks
DDI-26	atorvastatin or simvastatin or lovastatin + verapamil or diltiazem	PK		
DDI-27	simvastatin + amlodipine	PK		
DDI-28	atorvastatin or simvastatin or lovastatin + amiodarone	PK		
DDI-29	atorvastatin or simvastatin or lovastatin + some macrolides (i.e. erythromycin or clarithromycin or roxithromycin or telithromycin)	PK		
DDI-30	calcium channel blocker + a CYP3A4 inhibitor ‡	PK	Increased effects of calcium channel blockers	- Monitor patients for cardiotoxicity (e.g. QT prolongation, torsade de pointes, bradycardia, congestive heart failure)<br>- Advise patients to promptly report any increased effects of calcium channels blockers such as headache, flushing, excessive hypotension, reflex tachycardia, oedema, difficulties breathing, chest pain or tightness
DDI-31	disopyramide + some macrolides (i.e. erythromycin, clarithromycin telithromycin)	PK + PD	Hypoglycaemic coma, QT prolongation, torsade de pointes, heart block and ventricular fibrillation	- Avoid concurrent use except if benefits outweigh risks<br>- Substitute with non-macrolide antibiotic<br>- Closely monitor patients for cardiotoxicity<br>- Advise patients to promptly report any symptoms that could indicate the occurrence of torsade de pointes such as dizziness, light-headedness, fainting, palpitations, irregular heartbeat, shortness of breath, or syncope
DDI-32	beta-blocker + verapamil or diltiazem	PD	Potentially serious cardiovascular adverse effects including congestive	- Avoid concurrent use, particularly in patients predisposed to heart failure<br>- Closely monitor patient hemodynamic response and tolerance and adjust the dosage of one or both agents accordingly<br>- Advise patients to promptly report any symptoms including fatigue, headache, fainting, swelling of the extremities, weight gain, shortness of breath, chest pain, increased or decreased heartbeat, or irregular heartbeat

			heart failure, severe hypotension, exacerbation of angina, ventricular asystole, sinus arrest, heart block	
DDI-33	procainamide + amiodarone	PD	(Exacerbation of pre-existing) arrhythmias and QT prolongation	- Avoid concurrent use except for patients with life-threatening ventricular arrhythmias who are incompletely responsive to a single agent or to amiodarone alone<br>- Reduce the dosage of both agents by one-third to one-half<br>- Monitor patients for conduction disturbances and exacerbation of tachyarrhythmia<br>- Advise patients to promptly report any symptoms that could indicate the occurrence of torsade de pointes such as dizziness, light-headedness, fainting, palpitations, irregular heartbeat, shortness of breath, or syncope.
DDI-34	procainamide + trimethoprim	PK	Cardiac adverse effects including QT prolongation, torsade de pointes, cardiac arrest	- Reduce the procainamide dosage. Monitor serum procainamide levels as well as patient response and adjust the procainamide dosage accordingly<br>- Patients should be advised to promptly report any signs of procainamide toxicity including drowsiness, dizziness, syncope, confusion, tremor or palpitations
DDI-35	furosemide + etacrynic acid	PD	Ototoxicity with risk of tinnitus, reversible or irreversible hearing impairment, deafness	- Avoid concurrent use
DDI-36	concomitant use of ≥ 3 centrally-acting drugs (i.e. opiates or antipsychotics or benzodiazepines/z-drugs or barbiturates or antiepileptics or antidepressants)	PD	Increased risk of falls and fracture, impaired cognition	- Minimise the number of CNS agents<br>- Limit the dosage and duration of each drug to the minimum possible while achieving the desired clinical effect<br>- Closely monitor patients for adverse effects
DDI-37	alprazolam or diazepam or midazolam or triazolam or zolpidem or zopiclone + a	PK	Excessive sedation and prolonged	- Consider benzodiazepine/Z-drug dosage reduction<br>- Advise patients to promptly report any symptoms of nausea, vomiting, diarrhoea, confusion, daytime sedation, dizziness or unconsciousness

	CYP3A4 inhibitor ‡		hypnotic effects	
DDI-38	SSRI + another serotonergic drug (including tramadol)	PD<br><br>With tramadol: PD + PK	Serotonin syndrome<br><br>With tramadol: seizures and diminished therapeutic response to tramadol	- Closely monitor for symptoms of the serotonin syndrome such as hypertension, tachycardia, hyperthermia, myoclonus, mental status changes, particularly when initiating or increasing dosages of these agents. Consider potential risk even when administering serotonergic agents sequentially, as some of them may demonstrate prolonged elimination half-life (e.g. fluoxetine)<br>- With tramadol, use with caution regarding increased risk of seizure and monitor patient's therapeutic response<br>- When discontinuing a serotonergic CYP2D6 inhibitor in a patient receiving tramadol therapy, consider a tramadol dose reduction and monitor for signs of respiratory depression or sedation
DDI-39	oral NSAID + SSRI or SNRIs	PD	Bleeding, gastrointestinal bleeding	- Substitute with alternatives to NSAIDs (e.g. paracetamol) or less gastrotoxic NSAIDs (e.g. ibuprofen)<br>- Consider the addition of gastroprotective drugs (e.g. PPI, H_2 antagonists)<br>- Advise patients to promptly report any signs of excessive anticoagulation such as unusual or prolonged bleeding, bruising, vomiting, change in stool or urine colour, headache, dizziness or weakness
DDI-40	fluoxetine + tricyclic antidepressant	PD + PK	Serotonin syndrome<br><br>Tricyclic antidepressant toxicity, including cardiac arrhythmias	- Consider tricyclic antidepressant dosage reduction and serum level monitoring, even several weeks after fluoxetine discontinuation<br>- Closely monitor patients for signs of tricyclic antidepressants toxicity (e.g. cardiac arrhythmias, sedation, dry mouth, blurred vision, constipation, urinary retention) and/or excessive serotonergic activity (e.g. CNS irritability, altered consciousness, confusion, myoclonus, ataxia, abdominal cramping, hyperpyrexia, shivering, pupillary dilation, diaphoresis, hypertension, and tachycardia)<br>- If serotonin syndrome occurs, immediately discontinue fluoxetine and tricyclic antidepressants<br>- If ventricular arrhythmias develop, consider fluoxetine discontinuation and cardiac evaluation
DDI-41	lithium + NSAID	PK	Lithium toxicity, potentially life-threatening	- Extra caution is advised in a number of conditions including advanced age, impaired renal function, decreased sodium intake, volume depletion, renal artery stenosis, and heart failure as these increase the risk of toxicity.<br>- Reduce the lithium dosage, titrate slowly and frequently monitor serum concentrations<br>- Closely monitor patients for signs of lithium toxicity including drowsiness, dizziness, confusion, weakness, ataxia, tremor, tinnitus, blurred vision, nystagmus, vomiting, diarrhoea, thirst, diabetes insipidus (polyuria, polydipsia), seizure and ECG changes<br>- Advise patients to promptly report any signs of lithium toxicity (*listed above*)
DDI-42	lithium + diuretic	PK		
DDI-43	lithium + ACE inhibitor or an ARB	PK		
DDI-44	MAO inhibitor (i.e. phenelzine, moclobemide, rasagiline, safinamide, selegiline, linezolid) + sympathomimetic	PD	Hypertensive crisis	- DDI-44: Concurrent use is contraindicated. Wait at least 14 days after MAO inhibitor discontinuation before starting sympathomimetic use<br>- DDI-45: Avoid concurrent use even if carbidopa or benserazide are given in combination with levodopa. Wait two to three weeks after MAO-A inhibitor discontinuation before starting levodopa treatment

DDI-45	MAO-A inhibitor (i.e. moclobemide) or non-selective MAO inhibitor (i.e. phenelzine or linezolid) + levodopa	PD		
DDI-46	MAO inhibitor (i.e. phenelzine, moclobemide, rasagiline, safinamide, selegiline, linezolid) + some opioids (i.e. meperidine or fentanyl)	PD	Serotonin syndrome<br>Respiratory depression, cyanosis, hypotension and coma	- Concurrent use is contraindicated or not recommended<br>- Wait at least 14 days after MAO inhibitor discontinuation before starting an opioid
DDI-47	MAO inhibitor (i.e. phenelzine, moclobemide, rasagiline, safinamide, selegiline, linezolid) + an antidepressant (particularly a SSRI)	PD	Serotonin syndrome	- Concurrent use is contraindicated<br>- Wait at least 14 days between stopping a MAO inhibitor and starting another antidepressant; wait at least 7 to 14 days between stopping another antidepressant and starting a MAO inhibitor (5 weeks with fluoxetine)<br>- Monitor patients for signs of serotonin syndrome (hypertension, tachycardia, hyperthermia, myoclonus, mental status changes)
DDI-48	carbamazepine + verapamil or diltiazem	PK	Carbamazepine toxicity<br>Decreased therapeutic effect of verapamil, diltiazem, macrolide	- Closely monitor serum levels of carbamazepine and adjust the dosage accordingly<br>- Advise patients to promptly report any signs of carbamazepine toxicity such as headache, nausea, vomiting, dizziness, confusion, slurred speech, nystagmus, visual disturbances, tremors and ataxia<br>- DDI-48: Reduce carbamazepine dosage by one-half upon initiation of verapamil or diltiazem. Monitor blood pressure and cardiac effect after initiating carbamazepine<br>- DDI-49: Substitute with a non-macrolide antibiotic therapy or wait at least two weeks of discontinuing carbamazepine before using a macrolide. If co-administered, monitor patients for antimicrobial efficacy
DDI-49	carbamazepine + some macrolides (i.e. erythromycin or clarithromycin or roxithromycin or telithromycin)	PK		
DDI-50	acetylcholinesterase inhibitor + a drug that reduces heart rate (i.e. antiarrhythmic drugs or beta-blockers or verapamil or diltiazem)	PD	Bradycardia	- Use with caution, particularly in patients with increased risk of developing cardiac conduction disturbances<br>- Advise patients to promptly report any symptoms such as dizziness, light-headedness, fainting or irregular heartbeat
DDI-51	theophylline + cimetidine	PK	Theophylline toxicity	- Closely monitor the theophylline serum levels and adjust the dosage accordingly<br>- Advise patients to promptly report any signs of theophylline toxicity such as nausea, vomiting, diarrhoea, headache, seizures, restlessness, insomnia, or irregular heartbeat/palpitations<br>- DDI-54: Substitute with other SSRI or reduce the theophylline dosage by one-third and closely
DDI-52	theophylline + a quinolone	PK		

DDI-53	theophylline + some macrolides (i.e. erythromycin or clarithromycin or roxithromycin or telithromycin)	PK		monitor serum theophylline levels
DDI-54	theophylline + fluvoxamine	PK		
DDI-55	thiopurines (e.g. azathioprine) + allopurinol	PK	Azathioprine toxicity	- Reduce the azathioprine dosage by one-quarter to one-third<br>- Closely monitor patients for hematologic toxicity (leukopenia, thrombocytopenia, anaemia)<br>- Advise patients to report any signs of thiopurine toxicity such as fever, chills, sore throat, fatigue, lethargy, pallor, anorexia, jaundice, dark urine, nausea, vomiting, signs of local infection and unusual bleeding or bruising
DDI-56	oral or parenteral corticosteroid + an oral NSAID	PD	Gastrointestinal ulceration or bleeding	- Consider the addition of gastroprotective drugs (e.g. PPI, H_2 antagonists)<br>- Advise patients to report any signs of gastrointestinal ulceration and bleeding such as severe abdominal pain, dizziness, light-headedness and the appearance of black, tarry stools
DDI-57	concomitant prescription of ≥ 2 anticholinergic drugs	PD	Anticholinergic effects including cognitive decline	- Minimise the number of anticholinergic drugs and consider non-anticholinergic alternatives<br>- Closely monitor patients for additive anticholinergic effects such as mydriasis, blurred vision, flushed face, fever, dry skin and mucous membranes, tachycardia, urinary retention, constipation, memory loss, disorientation, incoherence, hallucinations, psychosis, delirium, hyperactivity, twitching or jerking movements, stereotypy and seizures<br>- Advise patients to promptly report any potential signs of anticholinergic effects such as abdominal pain, fever, heat intolerance, blurred vision, confusion or hallucinations
DDI-58	ciclosporin + rifampicin	PK	Organ rejection	- Monitor serum levels of the immunosuppressant and adjust the dosage accordingly<br>- Monitor patient for signs of organ rejection
DDI-59	ergot alkaloid (e.g. ergotamine) + some macrolides (i.e. erythromycin or clarithromycin or roxithromycin or telithromycin)	PK	Ergot toxicity	- Concurrent use is contraindicated given the potential for ergot toxicity characterised by nausea, vomiting, peripheral vasospasm, ischemia, thrombosis, tachycardia and hypertension
DDI-60	methotrexate + trimethoprim	PD + PK	Potentially fatal methotrexate toxicity	- Closely monitor patients for hematologic toxicity (e.g. myelosuppression, pancytopenia, megaloblastic anaemic, severe bone marrow depression)<br>- Advise patients to promptly report any signs and symptoms of bone marrow depression or anaemia such as fever, chills, sore throat, easy bruising or bleeding, pallor, dizziness, fatigue, lethargy, sore mouth or tongue and tingling in hands or feet
DDI-61	phosphodiesterase type 5-inhibitor + nitrate	PD	Severe hypotension, myocardial ischemia	- Concomitant use is contraindicated<br>- The time after when nitrates can be safely administered following PDE5 inhibitors use is uncertain and could go as far as 48 hours. Even then, closely monitor patients for hemodynamic response

DDI-62	tamoxifen + vitamin K antagonist	Unknown	Bleeding	- Concomitant use is contraindicated<br>- Consider using lower doses of vitamin K antagonist and closely monitor the INR
DDI-63	tamoxifen + citalopram or escitalopram	PD	Ventricular arrhythmias, torsade de pointes and sudden death	- Closely monitor patients for ECG changes<br>- Advise patients to promptly report any signs of toxicity such as drowsiness, dizziness, fainting/syncope, confusion or palpitations
DDI-64	tamoxifen + paroxetine or fluoxetine or bupropion	PK	Reduced effectiveness of tamoxifen	- Consider other antidepressant with a limited impact in CYP2D6 activity or, eventually, aromatase inhibitors as tamoxifen substitutes
DDI-65	concomitant prescription of ≥ 2 drugs that reduce potassium (e.g. β2-agonists, thiazides, loop diuretics, corticosteroids)	PD	Hypokalaemia, QT prolongation and torsade de pointes	- Closely monitor serum potassium levels<br>- Advise patients to promptly report any signs of hypokalaemia such as fatigue, weakness, myalgia, muscle cramps, numbness, tingling, abdominal pain, constipation, palpitation, and irregular heartbeat.
DDI-66	SSRI + loop or thiazide diuretic	PD	Hyponatraemia, orthostatic hypotension	- Closely monitor patients' sodium levels, blood pressure and pulse<br>- Advise patient to avoid rising abruptly from a sitting or recumbent position and to promptly report any signs of hyponatraemia including nausea, vomiting, headache, confusion, lethargy, weakness

Abbreviations: ACE: angiotensin-converting enzyme; ARB: angiotensin II type 1 receptor blockers; CNS: central nervous system; CrCl: creatinine clearance; CYP: cytochrome P450; DDI: drug-drug interaction; ECG: electrocardiogram; H2: histamine-2-receptor; INR: International Normalised Ratio; MAO: Monoamine oxidase; NSAID: non-steroidal anti-inflammatory drug; OATP: organic anion transporting polypeptide; PD: pharmacodynamic; PDE5: phosphodiesterase type 5; PK: pharmacokinetic; P-gp: P-glycoprotein; PPI: proton pump inhibitor; SmPC: Summary of product characteristics; SNRI: serotonin-norepinephrine reuptake inhibitor; SSRI: selective serotonin reuptake inhibitor

† Pharmacodynamic DDIs occur between drugs with additive or opposing effects. They can be anticipated based on knowledge of the clinical effects of the drugs involved (mode of action, organs affected in relation to action or side effects). Pharmacokinetic DDIs cannot be predicted from the clinical effects of the drugs involved. They require knowledge on the PK parameters (absorption, distribution, metabolism and elimination) of each drug, and these parameters may vary between drugs of the same pharmacological class.

‡ CYP3A4 inhibitors include ketoconazole, itraconazole, voriconazole, posaconazole, fluconazole, diltiazem, verapamil, quinidine, dronedarone, amiodarone, ciclosporin, ritonavir, clarithromycin, erythromycin

1. Anrys P, Petit A-E, Thevelin S, et al. An International Consensus List of Potentially Clinically Significant Drug-Drug Interactions in Older People. *J Am Med Dir Assoc*. Published online April 23, 2021. doi:10.1016/j.jamda.2021.03.019

Additional file 2. Classification of drug-drug interactions (DDIs) according to their potential harm

- Serious cardiovascular adverse effects : hypoakaemia, cardiac arrhythmia (including QT prolongation, torsade de pointes, heart block and ventricular fibrillation), congestive heart failure, exacerbation of angina, altered blood pressure (hypotension or hypertension) [includes increased effects of calcium channel blockers]
- Serious neurologic adverse effects : Serotonin syndrome, increased risk of falls and fracture, impaired cognition, excessive sedation and prolonged hypnotic effects, lithium toxicity, carbamazepine toxicity, hypoglycaemic coma, anticholinergic effects
- Bleeding
- Deterioration of renal function and / or hyperkalaemia (including severe myopathy and rhabdomyolysis which may lead to acute renal failure)
- Hematologic toxicity: azathioprine toxicity, methotrexate toxicity
- Others

**Potential harm**	**List of drug-drug interactions**
Serious cardiovascular adverse effects	DDI1, DDI2, DDI3, DDI4, DDI5, DDI6, DDI30, DDI31, DDI32, DDI33, DDI34, DDI44, DDI45, DDI50, DDI61, DDI63, DDI65
Serious neurologic adverse effects	DDI36, DDI37, DDI38, DDI40, DDI41, DDI42, DDI43, DDI46, DDI47, DDI48, DDI49, DDI57
Bleeding	DDI7, DDI8, DDI9, DDI10, DDI11, DDI12, DDI13, DDI14, DDI15, DDI16, DDI17, DDI18, DDI19, DDI20, DDI39, DDI56, DDI62
Deterioration of renal function and / or hyperkalaemia (including severe myopathy and rhabdomyolysis which may lead to acute renal failure)	DDI21, DDI22, DDI23, DDI24, DDI25, DDI26, DDI27, DDI28, DDI29, DDI30
Hematologic toxicity	DDI55, DDI60,
Others	DDI35, DDI51, DDI52, DDI53, DDI54, DDI58, DDI59, DDI64, DDI66

Additional file 3. International Classification of Diseases, 10th revision (ICD-10) codes used to identify comorbid conditions during the index hospitalization

	**ICD10 codes**
Dementia	F00; F01; F02; F03; F05.1; G30; G31.1
Depression	F32; F33
Stroke	I63.X;I69;X;I74.X;G45.X;G46.X
Hypertension	I10.X; I15.X
Diabetes	E10.X–E14.X; G590; G632; G730; G990; H280; H360; I792; L97; M142; M146; N083; G590; G632; G730; G990; H280; H360; I792; L97; M142; M146; N083
Non-valvular atrial fibrillation	I48.X
Coronary heart disease	I20.X-I25.X
Heart failure	I11.0; I13.0; I13.2; I13.9; I50.X; K76.1; J81.X
Chronic renal failure	N18.X; I12.X; I13.1; I13.2; E10.2; E11.2; E13.2; E14.2;N08.3; Z49.0-Z49.2; Z94.0; Z99.2
Chronic hepatic disease	R18.X; I85.X; K70.X; K71.4; K71.5; K71.7; K72.X; K73.X; K74.X; K76.1; B18.X; C22.X; C78.7
COPD	J43.X; J44.X
Cancer	C00.X-C26.X;C30.X-C34.X;C37.X-C41.X;C43.X; C45.X-C58.X; C60.X-C76.X;C81.X-C85.X;C88.X; C90.X-C97.X; C77.X-C80.X
History of hospitalization for major bleeding	I60.X-I62.X; S063; S064; S065; S066; K250; K252; K254; K256; K260; K262; K264; K266; K270; K272; K274; K276; K280; K282; K284; K286; K290; K920; K921; K922; I850; N02; R31; J942; R040; R041; R042; R048; R049; D62.X; K661; K624; M250; R58.X; N920; N921; N924; N938; N939; N920; N950; H113; H356; H431; H450; H922; I312
Venous thrombo-embolism	I26.X; I80.X-I82.X

Abbreviations: COPD: chronic obstructive pulmonary disease

Additional file 4. Prevalence (%) of all drug-drug interactions (DDIs) over time

**DDI**	**Baseline**	**Discharge**	**Month 2**	**Month 6**	**Month 12**
**At least one**	**53.6**	**58.3**	**56.9**	**56.4**	**56.8**
DDI-1	0.1	0.1	0.1	0	0
DDI-2	0.2	0.2	0.2	0.1	0
DDI-3	0	0	0	0	0
DDI-4	0	0	0	0	0
DDI-5	0.2	0.1	0.1	0.1	0.1
DDI-6	2.6	3.2	2.6	2.2	2.1
DDI-7	0.1	0.1	0.1	0	0
DDI-8	0	0	0	0	0
DDI-9	0	0	0	0	0
DDI-10	1.3	1.4	1.3	1	1
DDI-11	5.1	5.5	5.6	5.7	5.6
**DDI-12**	**5.7**	**7.2**	**7.5**	**7**	**7**
DDI-13	0.2	0.3	0.3	0.3	0.3
DDI-14	0.2	0.6	0.1	0.1	0
DDI-15	0.3	0.3	0.2	0.3	0.3
DDI-16	0.2	0.2	0.2	0	0.2
DDI-17	0.1	0.1	0.1	0.1	0.1
DDI-18	1.4	1.3	1.5	1.6	1.4
DDI-19	1.1	1.5	1.2	1.5	1.4
DDI-20	1.6	1.3	1.6	2.5	2.1
**DDI-21**	**10.3**	**10.9**	**11.8**	**12.8**	**12.6**
DDI-22	1.1	2.2	1.8	0.9	0.5
DDI-23	2.6	1.7	2	3.2	3.2

DDI-24	2.2	1.2	1.6	2.1	2.8
DDI-25	0.1	0.1	0.1	0.1	0.1
DDI-26	0.7	0.7	0.6	0.6	0.6
DDI-27	3.1	2.9	2.9	2.9	2.8
DDI-28	2	2.2	2.1	1.9	2.1
DDI-29	0.1	0.3	0	0	0.1
DDI-30	2.6	2.6	2	2.1	2
DDI-31	0	0	0	0	0
DDI-32	0.4	0.6	0.5	0.4	0.3
DDI-33	0	0	0	0	0
DDI-34	0	0	0	0	0
DDI-35	0	0	0	0	0
**DDI-36**	**10.8**	**11.9**	**13**	**12**	**13.4**
DDI-37	0.2	0.3	0.1	0.3	0.3
DDI-38	2.7	2.5	2.5	2.6	2.8
**DDI-39**	**6.1**	**5.2**	**5.6**	**6.4**	**6.3**
DDI-40	0.1	0.1	0.1	0.1	0.1
DDI-41	0.2	0.2	0.1	0.1	0.1
DDI-42	0.2	0.1	0.1	0.1	0.1
DDI-43	0.2	0.2	0.2	0.2	0.1
DDI-44	0.1	0.2	0.1	0.1	0.1
DDI-45	0	0	0	0	0
DDI-46	0	0	0	0	0
DDI-47	0.1	0.1	0	0	0
DDI-48	0.1	0.1	0	0	0
DDI-49	0.1	0	0	0	0
DDI-50	0.8	0.6	0.6	0.8	0.6
DDI-51	0	0	0	0	0

DDI-52	0.2	0.2	0	0.1	0.1
DDI-53	0	0	0	0	0
DDI-54	0	0	0	0	0
DDI-55	0	0	0	0	0
DDI-56	4.6	4.8	4.9	4.6	4.1
DDI-57	0.7	0.9	0.8	0.8	0.5
DDI-58	0	0	0	0	0
DDI-59	0	0	0	0	0
DDI-60	0.1	0.1	0.1	0.1	0.1
DDI-61	0.1	0	0.1	0.1	0.1
DDI-62	0.1	0.1	0.1	0.1	0
DDI-63	0.1	0.1	0.1	0.1	0.1
DDI-64	0	0	0	0	0
**DDI-65**	**20**	**25.9**	**23.7**	**23.8**	**23.7**
DDI-66	4.7	5	4.9	5.2	5.4

All DDIs are detailed in **Additional file 1**

Additional file 5. The three most common drug-drug interactions (DDIs) in this cohort and medication classes involved

**Patients with at least this DDI at discharge*** **N = 1950**	**Most common drugs (ATC class) involved in these DDIs**
DDI 65 : concomitant prescription of ≥ 2 drugs that reduce potassium	
N = 505 (25.9%)	- C03C (high ceiling diuretics) : N = 359 (71%)<br>- R03AC (B2 agonists inhalants) : N = 273 (54%)<br>- H02 (corticosteroids systemic) : N = 190 (38%)<br>- A06AB (contact laxatives) : N =166 (33%)
DDI 36 : concomitant use of ≥ 3 centrally-acting drugs	
N = 232 (11.9%)	- N05 (antipsychotics, anxiolytics, hypnotics and sedatives) : N = 183 (79%)<br>- N06A (antidepressants) : N = 156 (67%)<br>- N02A (opioids) : N = 151 (65%)<br>- N03 (antiepileptics) : N = 122 (53%)
DDI 21 : concomitant use of ≥ 2 potassium-sparing drugs	
N = 223 (10.9%)	- C09 (agents acting on the renin-angiotensin system): N = 206 (92%)<br>- C03DA01 (spironolactone) : N = 130 (58%)

* discharge = end of the index hospitalization

Abbreviation: ATC: Anatomical Therapeutic Chemical, DDIs: drug-drug interactions

Additional file 6. Prevalence of drug-drug interactions (DDIs) during follow-up (at least one DDI (“whole”) and DDIs belonging to the 3rd most frequent quartile (prevalence ≥ 1.5%)). A: in the experimental arm, B: in the control arm

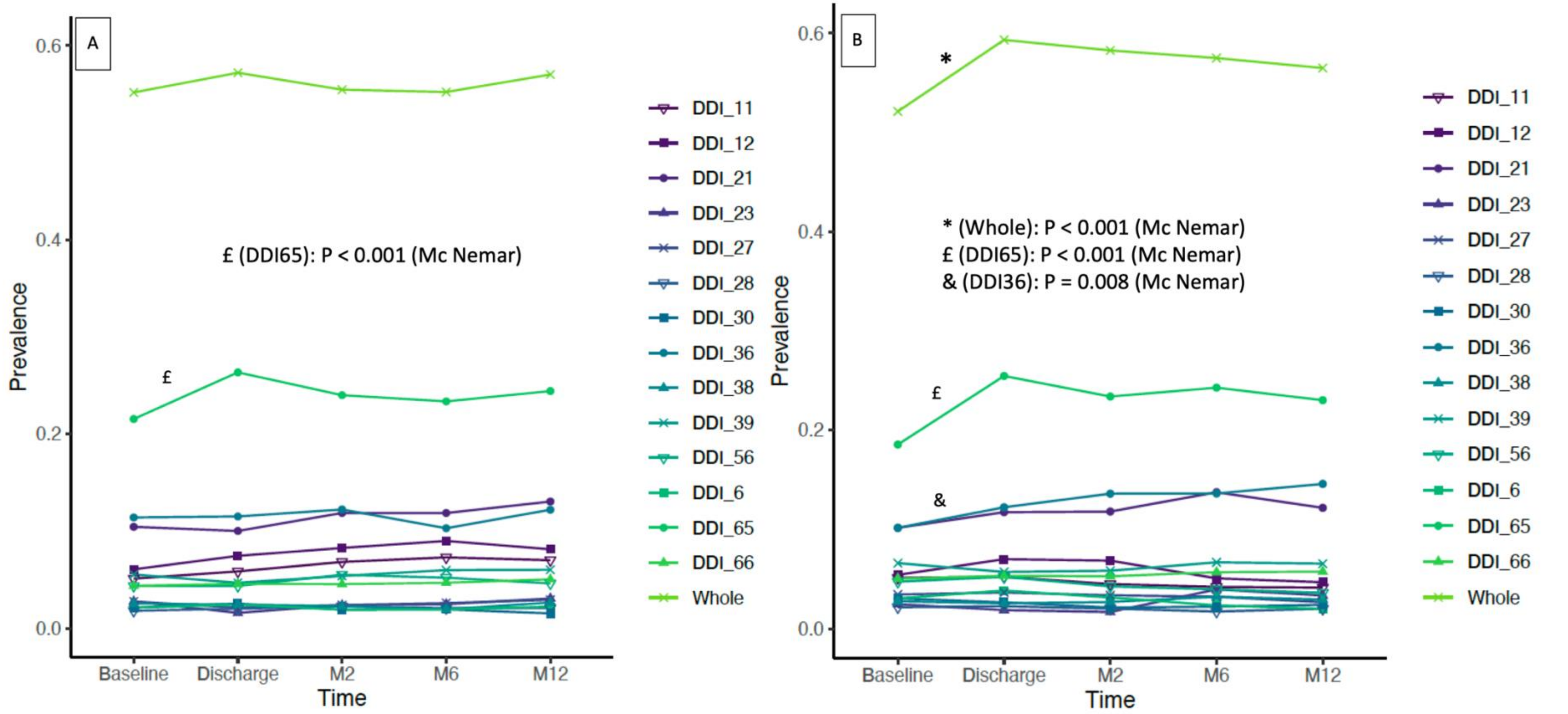

DDIs are detailed in Additional file 1 DDI 11: oral anticoagulant + an oral NSAID; DDI 12: oral anticoagulant + an antiplatelet drug; DDI 21: concomitant use of ≥ 2 potassium-sparing drugs; DDI 23: ACE inhibitor or ARB + an oral NSAID; DDI 27: simvastatin + amlodipine; DDI 28: atorvastatin or simvastatin or lovastatin + amiodarone; DDI 30: calcium channel blocker + a CYP3A4 inhibitor; DDI 36: concomitant use of ≥ 3 centrally-acting drugs; DDI 38: SSRI + another serotonergic drug; DDI 39: oral NSAID + SSRI or SNRIs; DDI 56: oral or parenteral corticosteroid + an oral NSAID; DDI 6: digoxin + thiazide or loop diuretic; DDI 65: concomitant prescription of ≥ 2 drugs that reduce potassium; DDI 66: SSRI + loop or thiazide diuretic

Abbreviations: ACE: angiotensin-converting enzyme; ARB: angiotensin II type 1 receptor blockers; CNS: central nervous system, CYP: cytochrome P450; DDI: drug-drug interaction, NSAID: non-steroidal anti-inflammatory drug, M: month, PPI: proton pump inhibitor, SNRI: serotonin-norepinephrine reuptake inhibitor; SSRI: selective serotonin reuptake inhibitor

Additional file 7. Prevalence of drug-drug interactions (DDIs) during follow-up: at least one DDI for the whole cohort (“whole”) and according to site

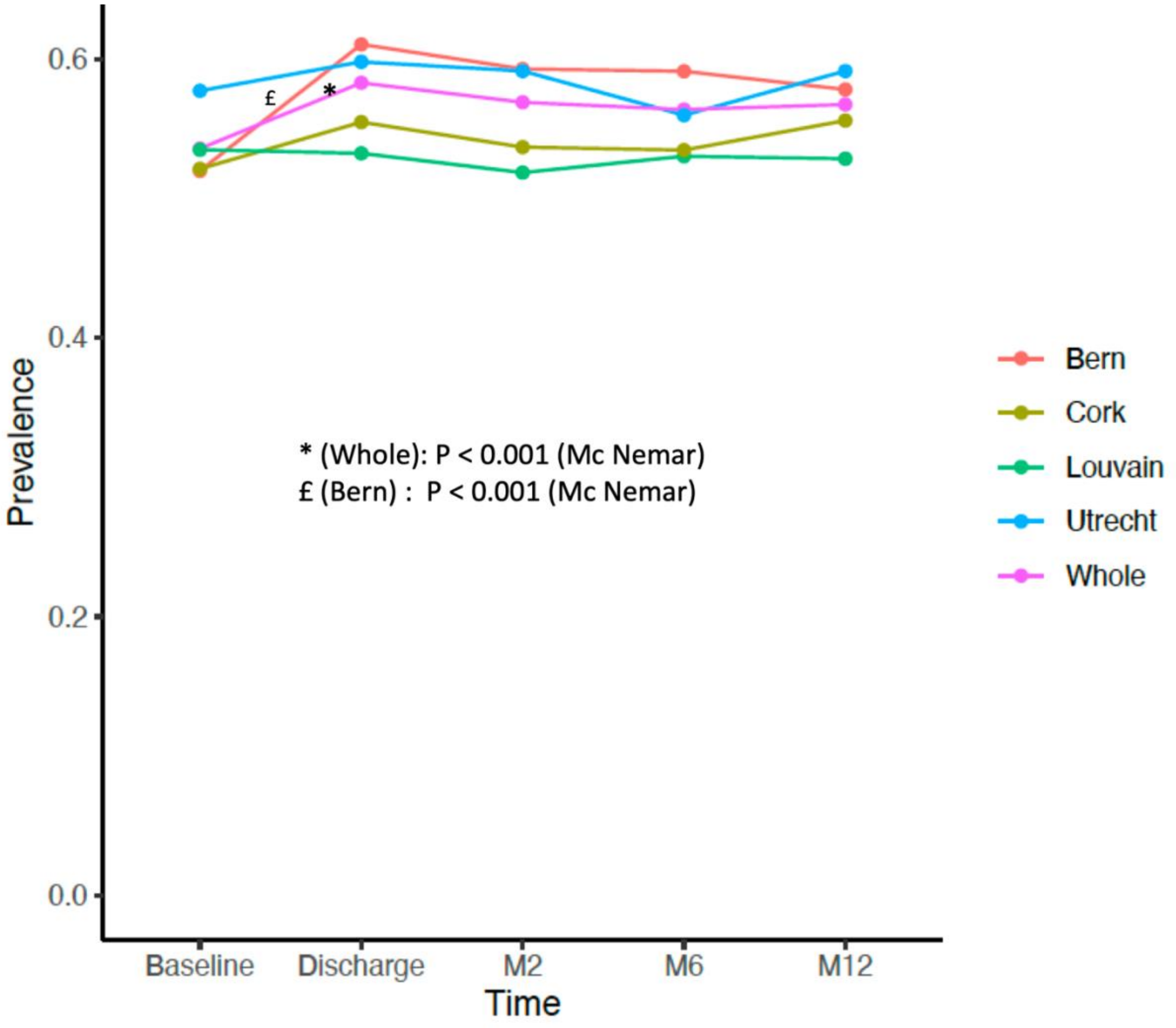

McNemar’s test was used to estimate differences in prevalence between baseline and discharge. Chi-squared test for trend was used to estimate the prevalence trend between discharge and one year after the inclusion. Only p value ≤ 0.05 are specified, other p values are > 0.5

Additional file 8. Demographic data and baseline characteristics of patients included in the cohort and stratified by site

	Total<br>N=1950	Bern<br>N=804	Louvain<br>N=385	Utrecht<br>N=433	Cork<br>N=328	p	Missing values
**Age (years)**							0
Median (IQR)	79 [74 – 84]	79 [74 – 84]	78 [73 – 83]	78 [74 – 83]	80 [75 – 85]		
70 – 79	1053 (54)	424 (53)	222 (58)	253 (58)	154 (47)	<.001	
80– 89	757 (39)	313 (39)	146 (38)	149 (35)	149 (45)	.008	
> 90	140 (7)	67 (8)	17 (4)	31 (7)	25 (8)		
**Male sex**	1080 (55)	464 (58)	208 (54)	248 (57)	160 (49)	.04	0
**Medical History**							0
Dementia	116 (6)	88 (11)	4 (1)	5 (1)	19 (6)	<.001	
Depression	173 (9)	94 (12)	22 (6)	20 (5)	37 (11)	<.001	
Stroke	410 (21)	208 (26)	29 (8)	103 (24)	70 (21)	<.001	
Hypertension	1282 (66)	530 (66)	290 (75)	247 (57)	215 (66)	<.001	
Diabetes	628 (32)	252 (31)	129 (33)	150 (35)	97 (30)	.42	
Atrial fibrillation	697 (36)	294 (37)	119 (31)	145 (33)	139 (42)	.01	
Coronary artery disease	661 (34)	314 (39)	124 (32)	125 (29)	98 (30)	.001	
Heart failure	500 (26)	241 (30)	80 (21)	108 (25)	71 (22)	.001	
Chronic renal failure	522 (27)	315 (39)	100 (26)	31 (7)	76 (23)	<.001	
Chronic hepatic failure	98 (5)	54 (7)	27 (7)	9 (2)	8 (2)	<.001	
COPD	375 (19)	124 (15)	47 (12)	136 (31)	68 (21)	<.001	
Cancer	482 (25)	204 (25)	67 (17)	159 (37)	52 (16)	<.001	
Bleeding	250 (13)	160 (20)	18 (5)	37 (9)	35 (11)	<.001	
Thromboembolic disease	236 (12)	124 (15)	48 (12)	30 (7)	34 (10)	<.001	
Number of comorbidities	11 [8 – 16]	16 [11 – 21]	9 [7 – 13]	8 [6 – 10]	10 [8 – 13]	<.001	
Number of drugs per day	12 [9 – 16])	14 [11 – 18]	10 [8 – 13]	12 [10 – 16]	11 [9 – 14]	<.001	0
Hyperpolypharmacy*	1458 (75)	676 (84)	218 (57)	337 (78)	227 (69)	<.001	0
**Any hospitalisations during the last year**	986 (51)	410 (51)	183 (48)	226 (52)	167 (51)	.58	0
**Non-independently living**†	375 (19)	92 (11)	138 (36)	68 (16)	77 (23)	<.001	0

	Total N=1950	Bern N=804	Louvain N=385	Utrecht N=433	Cork N=328	p	Missing values
**Type of admission**‡						<.001	0
Elective	73 (24)	137 (17)	204 (53)	117 (27)	15 (5)		
Non elective	1477 (76)	667 (83)	181 (47)	316 (76)	313 (95)		

* ≥ 10 drugs par day at admission

† Non-independently living was defined as living in a nursing home (at least 3 months in the 6 months before the index admission) or being housebound

‡ Elective procedure for a pre-existing condition

Data are Median [P25; P75] or n (%). Comparison between the two groups by Mann-Whitney U test for quantitative variables and chi-square test or Fisher's exact test for qualitative variables.

Abbreviations: COPD: chronic obstructive pulmonary disease

Additional file 9. Demographic data and baseline characteristics of older patients present at 2 months and stratified by DDI decrease status at 2 months compared to baseline*

	Total N = 1722	At least one decrease in DDI at 2 months N = 331	No decrease in DDI at 2 months N = 1391	Missing values	*P* value
**Age (years)**					
Median (IQR)	78 [74 – 84]	79 [74 – 84]	78 [74 – 84]	0	.50
70 – 79	943 (55)	172 (52)	771 (56)		.49
80– 89	669 (39)	138 (42)	531 (38)		
> 90	110 (6)	21 (6)	89 (6)		
**Male sex**	960 (56)	167 (51)	793 (57)	0	.06
**Trial site**					
Louvain, Belgium	322 (19)	64 (19)	258 (19)	0	.15
Cork, Ireland	298 (17)	43 (13)	255 (18)		
Utrecht, The Netherlands	355 (21)	73 (22)	282 (20)		
Bern, Switzerland	747 (43)	151 (46)	596 (43)		
**Randomization arm:**					
Experimental	833 (48)	168 (51)	665 (48)	0	.37
Control	889 (52)	163 (49)	726 (52)		
**Medical history**					
Dementia	106 (6)	23 (7)	83 (6)	0	.58
Depression	152 (9)	43 (13)	109 (8)		.04
Stroke	363 (21)	75 (23)	288 (21)		.48
Hypertension	1132 (66)	217 (66)	915 (66)		.99
Atrial fibrillation	610 (35)	138 (42)	472 (34)		.01
Diabetes	554 (32)	108 (33)	446 (32)		.89
Coronary artery disease	583 (34)	124 (37)	459 (33)		.14
Heart failure	437 (25)	111 (33)	326 (23)		<.001
Chronic renal failure	477 (28)	97 (29)	380 (27)		.51
Chronic hepatic failure	81 (5)	15 (5)	66 (5)		.98

Continuation of table

	Total N = 1722	At least one decrease in DDI at 2 months N = 331	No decrease in DDI at 2 months N = 1391	Missing values	*P* value
**Medical history**					
COPD	325 (19)	74 (22)	251 (18)	0	.08
Cancer	413 (24)	67 (20)	346 (25)		.09
Bleeding	224 (13)	43 (13)	181 (13)		.99
Thromboembolic disease	102 (6)	17 (5)	85 (6)		.58
Number of comorbidities	11 [8 – 16]	12 [8 – 16]	11 [8 – 15]		.01
**Medications on index admission**					
Hyperpolypharmacy†	1294 (75)	284 (86)	1010 (73)	0	< .001
**Non-independently living‡**	303 (18)	62 (19)	241 (17)	0	.91
**ADL6 score**	5.5 [4.5 – 6.0]	5.5 [4.0 – 6.0]	5.5 [4.5 – 6.0]	21	.61
**Education level**					
Less than high school education	491 (29)	98 (30)	393 (29)	20	.72
High-school degree	792 (46)	153 (47)	639 (46)		
Post-secondary degree	419 (25)	75 (23)	344 (25)		
**Type of admission§**					.71
Elective	417 (24)	77 (23)	340 (24)		
Non elective	1305 (76)	254 (77)	1051 (76)		
**Main reason for hospital admission**					.51
Surgery	230 (13)	40 (12)	190 (14)		
Medicine	1492 (87)	291 (88)	1201 (86)		

* Baseline = Index hospitalization

† ≥ 10 drugs par day at admission

‡ Non-independently living was defined as living in a nursing home (at least 3 months in the 6 months before the index admission) or being housebound

§ Elective procedure for a pre-existing condition

Data are Median [P25; P75] or n (%). Comparison between the two groups by Mann-Whitney U test for quantitative variables and chi-square test or Fisher's exact test for qualitative variables. Abbreviations: ADL: autonomy daily living; COPD: chronic obstructive pulmonary disease

Additional file 10. Demographic data and baseline characteristics of older patients included in our cohort and stratified by DDI increase status at 2 months compared to baseline*

	Total N = 1722	At least one increase in DDI at 2 months N = 459	No increase in DDI at 2 months N = 1263	Missing values	*P* value
**Age (years)**					
Median (IQR)	78 [74 – 84]	79 [74 – 83]	78 [74 – 84]	0	.97
70 – 79	943 (55)	255 (55)	688 (55)		.33
80– 89	669 (39)	169 (37)	500 (40)		
> 90	110 (6)	35 (8)	75 (6)		
**Male sex**	960 (56)	262 (57)	698 (55)	0	.53
**Trial site**					
Louvain, Belgium	322 (19)	65 (14)	257 (20)	0	< .001
Cork, Ireland	298 (17)	60 (13)	238 (19)		
Utrecht, The Netherlands	355 (21)	84 (18)	271 (22)		
Bern, Switzerland	747 (43)	250 (55)	497 (39)		
**Randomization arm:**					
Experimental	833 (48)	213 (46)	620 (49)	0	.35
Control	889 (52)	246 (54)	643 (51)		
**Medical history**					
Dementia	106 (6)	37 (8)	69 (5)	0	.06
Depression	152 (9)	50 (11)	102 (8)		.08
Stroke	363 (21)	107 (23)	256 (20)		.17
Hypertension	1132 (66)	289 (63)	843 (67)		.16
Atrial fibrillation	610 (35)	228 (50)	382 (30)		< .001
Diabetes	554 (32)	159 (35)	395 (31)		.21
Coronary artery disease	583 (34)	187 (41)	396 (31)		< .001
Heart failure	437 (25)	178 (39)	259 (21)		< .001
Chronic renal failure	477 (28)	146 (32)	331 (26)		.02
Chronic hepatic failure	81 (5)	19 (4)	62 (5)		.59

Continuation of table

	Total N = 1722	At least one increase in DDI at 2 months N = 459	No increase in DDI at 2 months N = 1263	Missing values	*P* value
**Medical history**					
COPD	325 (19)	94 (20)	231 (18)	0	.34
Cancer	413 (24)	102 (22)	311 (25)		.33
Bleeding	224 (13)	81 (18)	143 (11)		.001
Thromboembolic disease	102 (6)	25 (5)	77 (6)		.70
Number of comorbidities	11 [8 – 16]	12 [9 – 18]	10 [8 – 15]		< .001
**Medications on index admission**					
Hyperpolypharmacy†	1294 (75)	390 (85)	904 (72)	0	< .001
**Non-independently living‡**	303 (18)	81 (18)	222 (18)	0	.99
**ADL6 score**	5.5 [4.5 – 6.0]	5.5 [4.5 – 6.0]	5.5 [4.0 – 6.0]	21	.02
**Education level**					
Less than high school education	491 (29)	126 (28)	365 (29)	20	.35
High-school degree	792 (46)	230 (50)	562 (45)		
Post-secondary degree	419 (25)	99 (22)	320 (26)		
**Type of admission§**					.006
Elective	417 (24)	89 (19)	328 (26)		
Non elective	1305 (76)	370 (81)	935 (74)		
**Main reason for hospital admission**					.004
Surgery	230 (13)	43 (9)	187 (15)		
Medicine	1492 (87)	416 (91)	1076 (85)		

* Baseline = Index hospitalization

† ≥ 10 drugs par day at admission

‡ Non-independently living was defined as living in a nursing home (at least 3 months in the 6 months before the index admission) or being housebound

§ Elective procedure for a pre-existing condition

Data are Median [P25; P75] or n (%). Comparison between the two groups by Mann-Whitney U test for quantitative variables and chi-square test or Fisher's exact test for qualitative variables. Abbreviations: ADL: autonomy daily living, COPD: chronic obstructive pulmonary disease
